# Current State of SLC and ABC Transporters in the Skin and Their Relation to Sweat Metabolites and Skin Diseases

**DOI:** 10.3390/proteomes9020023

**Published:** 2021-05-16

**Authors:** Marcus M. K. Nielsen, Eva Aryal, Elnaz Safari, Biljana Mojsoska, Håvard Jenssen, Bala Krishna Prabhala

**Affiliations:** 1 Institute of Physics, Chemistry and Pharmacy, University of Southern Denmark, Campusvej 55, 5230 Odense, Denmark; marcn17@student.sdu.dk (M.M.K.N.); evary19@student.sdu.dk (E.A.); 2 Department of Organic Chemistry, Faculty of Chemistry, Urmia University, Urmia 5756151818, Iran; safarielnaz4@gmail.com; 3 Institute of Science and Environment, Roskilde University, Universitetsvej 1, 4000 Roskilde, Denmark; Biljana@ruc.dk (B.M.); jenssen@ruc.dk (H.J.)

**Keywords:** sweat metabolites, ABC transporters, SLC transporters, skin diseases, skin transporters, KNIME

## Abstract

With a relatively large surface area (2 m^2^) and 15% of total body mass, the skin forms the largest organ of the human body. The main functions of the skin include regulation of body temperature by insulation or sweating, regulation of the nervous system, regulation of water content, and protection against external injury. To perform these critical functions, the skin encodes genes for transporters responsible for the cellular trafficking of essential nutrients and metabolites to maintain cellular hemostasis. However, the knowledge on the expression, regulation, and function of these transporters is very limited and needs more work to elucidate how these transporters play a role both in disease progression and in healing. Furthermore, SLC and ABC transporters are understudied, and even less studied in skin. There are sparse reports on relation between transporters in skin and sweat metabolites. This mini review focuses on the current state of SLC and ABC transporters in the skin and their relation to sweat metabolites and skin diseases.

## 1. Introduction

**Figure 1:**
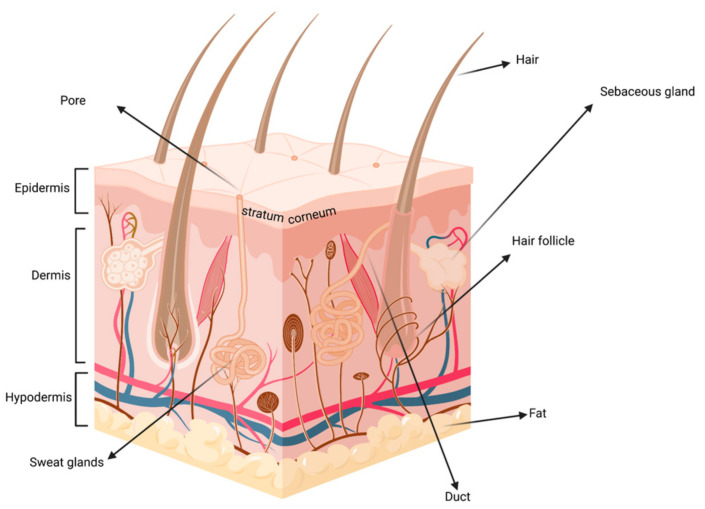
Schematic representation of skin. Created in biorender.com (accessed on 29 March 2021).

Skin is the largest organ of the body. It is usually soft, flexible and contains multiple layers, i.e., the stratum corneum, epidermis, dermis, and subcutaneous tissue and a myriad of cells with different functions (Figure 1). The skin comprises of two barriers that protect the body from environmental factors: One physical (stratum corneum) and one biochemical (epidermis and dermis). Nerves, glands, hair follicles, ducts, and blood vessels are embedded beneath the surface of the skin.

The skin has a well-established role in regulating body temperature by preventing excessive water loss through the stratum corneum [1] and by excreting water in the form of sweat. Sweat is produced by sweat glands in the dermis and reaches the epithelial surface of the skin through tiny ducts. Sweat glands are involved in perspiration and act as an excretory organ not only to efflux water and dilute electrolyte solution but also drugs and their metabolites [2]. There are three different types of sweat glands: Eccrine, apocrine, and apoeccrine. Eccrine sweat glands are extensively distributed across most of the body’s surface area and mainly secrete water and electrolytes. They directly open onto the skin surface by a duct and are responsible for large amounts of sweat production. Apocrine glands usually develop in association with hair follicles and open into them and secrete oily substances containing lipids, proteins, sugars, and ammonia through hair canals and are found only in skin containing hair such as the axilla, face, mammary, and genital areas [3,4]. Rather than responding to temperature, apocrine glands often respond to emotional stimulus, including anxiety, pain, and fear, and under these circumstances, sweating is often observed in the armpits, palms, and soles of the feet [3,4]. Apocrine glands are distributed in the same areas as apocrine glands but secrete watery fluids similar to eccrine glands [2]. Apoeccrine sweat glands are not widely reported. Like apocrine glands, they are limited to a specific area of the skin, i.e., only in the axillary area. Like eccrine glands, they secrete water and electrolytes directly onto the skin [5,6]. These glands may be useful in determining the secondary functions of sweat as evaporation is ineffective in the axillary region [6,7].

Sweat is normally a transparent body fluid with low tonicity and a slightly acidic nature with mean pH 6.3 [8]. Additionally, sweat metabolites are used as biomarkers for a variety of diseases [9]. Water is the main component of sweat ∼99% [8]. Moreover, sweat comprises a mixture of many chemicals in varying concentrations, including metal and nonmetal ions (e.g., Na+, K+, Ca2+, Mg2+, Fe2+, Zn2+, Cl−, and vitamins) as well as metabolites such as glucose, pyruvate, lactate, ammonia, urea, bicarbonate, amino acids, ethanol, cytokines, xenobiotics, betaine, and cortisol. The amount of Na+ and Cl− is the highest, ranging approximately from 10 mmol/L up to 90 mmol/L. Other compounds are present at a lower millimolar concentration (lactate, ethanol, urea, ammonia, bicarbonate, and K+), micromolar concentrations (Ca2+, Mg2+, Fe2+, Zn2+, Cu2+, ascorbic acid, glucose, and individual amino acids) or even smaller scale, nanomolar and picomolar concentrations (thiamine, cortisol, and cytokines) [7]. The efflux of electrolytes, such as Na+, K.+, Cl−, occurs through Na+–K+-ATPase, K+ and Cl- channels on the basolateral and apical membrane [10]. There is evidence that some membrane transport proteins are also responsible for both uptake and secretion of sweat metabolites, especially when it comes to metal ions [11].

**Figure 2:**
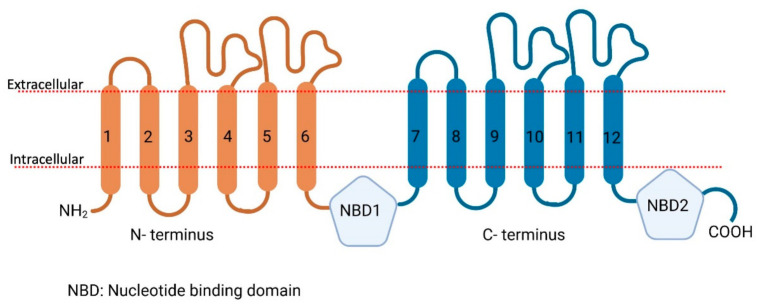
Schematic representation of the secondary structure of ABC transporters. N terminus in orange, C terminus in blue, NBD stands for nucleotide-binding domain. Created with biorender.com (accessed on 29 March 2021).

**Figure 3:**
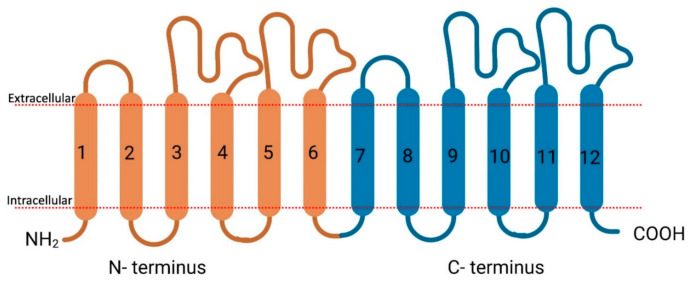
Schematic representation of the secondary structure of SLC transporters. N terminus in orange, C terminus in blue. Created with biorender.com (accessed on 29 March 2021).

Membrane transport proteins play an important role in the translocation of different endogenous and exogenous components (compounds, nutrients, metals, etc.) across plasma membranes [11]. Membrane transporters are widely distributed throughout the body, particularly in the epithelia of major organs, such as the liver, intestine, kidney, and other organs with barrier functions, such as the brain, skin, testes, and placenta [11,12,13,14,15,16]. Membrane transport proteins in humans are largely composed of two families: ATP-binding cassette (ABC) and solute carrier (SLC) transporters. ABC transporters are active transporters and utilize the energy of ATP binding (Figure 2) and hydrolysis to facilitate in- or efflux of their substrates across membranes [17,18,19] while on the other hand, SLC transporters (Figure 3) utilize ion electrochemical gradients, such as sodium or proton gradients for the uphill transport of the substrates [20].

As of now, 49 ABC transporters subtypes have been identified in humans, which are divided into 7 subfamilies ABCA, ABCB, ABCC, ABCD, ABCE, ABCF, and ABCG [19,21]. These transport a broad spectrum of endogenous substrates, including lipids (ABCA1 and ABCG family) [21,22,23,24], cholesterol (ABCG1) [25], iron (ABCB6–10) [26], organic anions (ABCC2), peptides (ABCB2 and 3), and bile salts (ABCB11) [27], as well as exogenous compounds, such as drugs across intra- and extracellular membranes [28]. In contrast, there are approximately 400 SLC transmembrane transporters that are divided into six groups. 66 distinct subfamilies (SLC1 to SLC66) have been identified [20,29].

**Figure 4:**
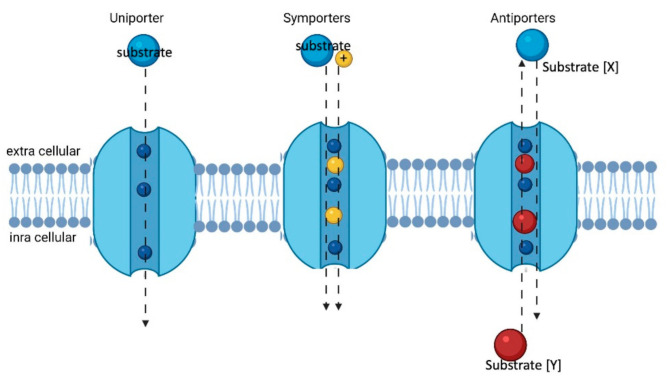
Schematic representation of the three types of SLC transporters. Created with biorender.com (accessed on 29 March 2021).

Like ABC family members, they transport various compounds ranging from the cellular uptake of nutrients to the absorption of xenobiotics [30]. Their function in the translocation of substrates can be described using four models: Cotransporter or symporter (SLC1 and SLC5), antiporters (SLC4 and SLC26), facilitated (SLC2) (Figure 4), and a few of them are ‘orphan transporter’ for which substrates and functions need to be identified [31]. In humans, ABC transporters mostly perform efflux functions by eliminating xenobiotics out of the body [32], while the SLC transport systems can be influxers, effluxers, or antiporters. A few SLC transporters have the ability for bidirectional transport [33] as well.

The present review will focus on the role of ABC and SLC transport proteins present in the skin and their correlation to sweat metabolites. A list of sweat metabolites was obtained from the human metabolome database (https://hmdb.ca/downloads accessed on 15 March 2021) [34]. Interestingly, very little evidence is available on the expression of different ABC and SLC transporters in the skin [35,36,37,38], and a thorough investigation is urgently needed to gain deeper insights into the role of SLC and ABC transporters in the skin and their relation to metabolites in sweat.

## 2. The Role of Proteomics in the Identification of Skin SLC and ABC Transporters

To investigate the role of transporters and other components of human skin, scientists have, for many years, used gene expression analysis. With the development of mass spectrometry (MS) analysis, we can now use MS-based proteomics approaches as a tool for quantification and identification of proteins in humans [39,40]. The advances in this technology have also allowed higher sensitivity when characterizing and analyzing large proteomes [41,42]. Knowledge about skin composition, especially the role of many transporters found in the skin, is limited. Recent studies have used proteomic investigation to try to capture the complexity of the skin [41]. The layered composition comprising of the inner and outer epidermis has been studied using in-depth MS-based proteomics utilizing both data-dependent acquisition and data-independent acquisition approaches. Using proteomics to investigate skin cell type diversity, including keratinocytes, fibroblasts, melanocytes, endothelial, and immune cells, revealed quantification of a total of 10,701 proteins and these findings have provided essential information for the unique systems and functions of each cell type within the skin complex [41].

Mass spectrometry-based proteomics uses label-free and label-based approaches. Label-free methods allow the identification of the abundance of many proteins, such as enzymes found in the human skin, and this knowledge is pivotal in our understanding of skin physiology and function. Most recently, for the first time, a label-free quantification of enzymes in human skin has been reported [43]. The study used this approach to compare proteins found in human skin and those in 3D models mimicking human skin. Here, ABC transporters ABCA8 and ABCB11 and eight SLC transporters (SLC12A2, SLC25A3, SLC25A5, SLC25A6, SLC29A1, SLC44A1, SLC44A2, SLC4A1) have been quantified in human skin along with xenobiotic-metabolizing enzymes (XME). ABC and other skin transporters regulate local drug concentrations in different tissues. Therefore, detection and quantification of their expression levels are important. Proteomics sample preparation can be challenging and especially when working with skin since this tissue contains a high lipid content and proteins with a high degree of crosslinking. It is even more challenging to quantify ABC transporters because they are large and low in abundance, which is close to the detection limit of liquid-chromatography tandem mass spectrometry (LC-MS/MS). ABC transporters found in the skin also share high homology (e.g., ABCB1, ABCB4, ABCB11, ABCA1, and ABCA2), and therefore, when analyzing these proteins, adjustments in the data analysis are necessary. For example, when publishing data sets, it is relevant to report the total intensities of unique peptides across all samples [38] along with the intensities of the shared peptides assigned according to the general ratio of the uniquely observed peptide intensities. Such analysis has been recently reported in a study where 32 ABC transporters across five tissues have been quantified [38].

Some of the sample preparation techniques include incubation in lysis buffer, homogenization, filtration, digestion, and sometimes enrichment [38,43]. Sample preparation plays an important role in the detection of these membrane proteins. Conventional methods such as 2D-gel electrophoresis did not result in significant protein yield [44]. Recently, different strategies have evolved, such as powdering the frozen tissues (from liquid nitrogen), and subsequent protein extraction has led to 10-fold increase in the number of identified proteins. Another strategy was an improvement to the existing tape stripping strategy in tandem with the state-of-the-art SP3 (single-pot solid phase enhanced sample preparation) approach [45,46]. This has led to an increase in the number of identified peptides. Different labs have different procedures, and the current state is that several methods should be used in tandem for lysing cells, extraction of proteins (more thorough screening of detergents is needed to extract and enrich membrane proteins), and purification of the tryptic digest.

## 3. Correlation between Sweat Metabolome and Skin SLC and ABC Transporters

We made a list of transport proteins characterized in the skin and tried to identify their substrates present in the sweat metabolites. Several ABC transporters are expressed in the skin [36], although none of them has been implicated with the transport of sweat metabolites in human skin, to the best of our knowledge. To confirm the same, we have utilized an advanced data mining tool KNIME. KNIME stands for Konstanz information MinEr, which is freely downloadable via www.knime.org, with compatibility over different operating systems, including Linux, MacOS, and Windows. KNIME operates in the form of workflows consisting of different nodes. Nodes are a part of workflow that, when bolted together, can allow computational operations ranging from something as simple as transposing a table, to performing advanced machine learning algorithms at the click of a button. To create workflows, nodes can simply be dragged and dropped into the workflow environment. More than 10,000 nodes are available in the node repository. With little knowledge of programming, one can create custom nodes in standard scripting languages such as MATLAB, R, Python, PERL, and JAVA [47,48].

To understand if sweat metabolites are in any way related to skin SLC and ABC transporters, we have utilized a premade workflow *“Term co-occurrence heatmap*” (https://www.knime.com/term-coocurrence-heatmap-example accessed on 15 March 2021, Figure 5), modified using KNIME version 4.3.1.

The workflow uses the protein names of the compiled SLC and ABC genes listed in Table 1 as search terms on PubMed, and up to 100 abstracts of resulting articles are downloaded and saved in a temporary directory. The metabolites are then used as dictionary terms, to discover if the metabolites are mentioned in the abstracts. If metabolites and proteins are related, then those documents are tagged, and untagged documents are filtered. The tagged documents are traced back to the transporters used as a search term, and the co-occurrences can thus be determined. Finally, heatmaps illustrating co-occurrences between transporters and metabolites were prepared using R. To remove false positives, we have added a filter where we filtered the entries with less than five co-occurrences. The remaining entries were scaled between 0 (white; metabolite cooccurred with the transporter less than five times) and 1 (black; metabolite that cooccurred with a transporter most). All the gray scales were the fractions between 0–1.

**Figure 6:**
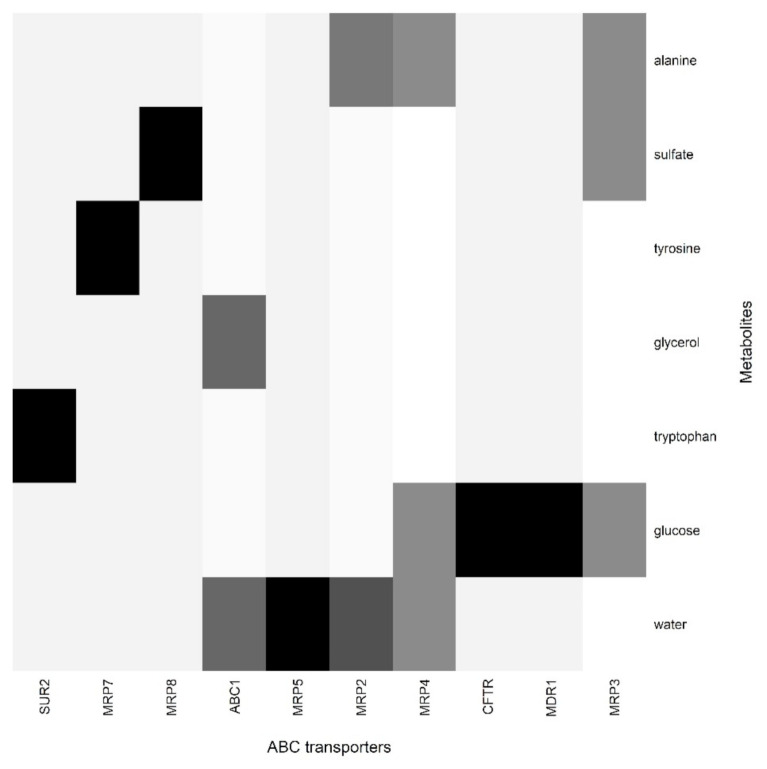
Co-occurrences of sweat metabolites with ABC transporter proteins.

**Figure 7:**
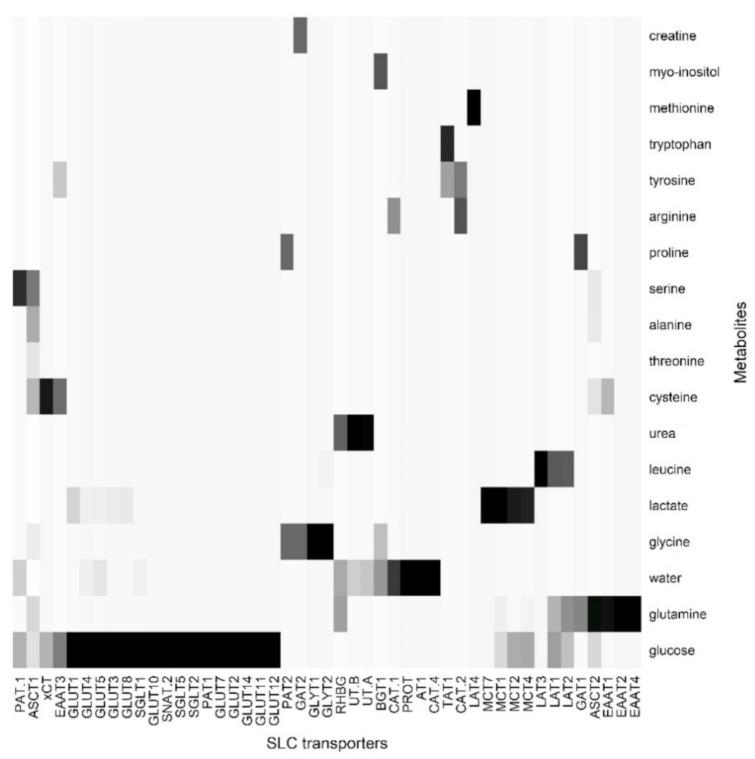
Co-occurrences of sweat metabolites with SLC transporter proteins.

This workflow gave output in the form of heatmaps (Figure 6 ABC transporter) and (Figure 7 SLC transporter).

Skin encodes for many ABC transporters, but very little evidence of co-relation between sweat metabolites and ABC transporters was observed. MDR1, also known as P-gp (ABCB1), MRP’s, SUR2 (ABCC9) [36,49,50,51], are expressed in the skin and are known to efflux a wide variety of drugs [32], but not much information on their real substrates is available.

For SLC transporters, we observed a stronger correlation with sweat metabolites. For example, SLC 36 transports glycine [52]. SLC7A1 and 2 are cationic amino acid transporters [53] and correlates well with arginine and ornithine. Similarly, SLC4 and 5 are glucose and bicarbonate transporters. They are multi-functional, allowing the passage for water channels and urea [54,55,56]. Glucose comprises one of the most well-known constituents of sweat. Glucose is the primary carbon source for the proliferative cells, four different SLC families have been detected to transport glucose, GLUT’s (SLC2A1–14), SGLT (SLC5), SWEET’s (SLC50), and SLC 60(A1–A2) of which GLUT’s have been observed to be expressed in the skin [57,58]. Lactate and pyruvates are also observed in sweat and these are generally transported by SLC16 family of transporters (MCT1–14) and several of them were observed to be expressed in skin [59]. Several amino acids were also observed in ‘faux sweat’ and skin encodes for many amino acid transporters such as SLC7 and SLC36 [60,61,62]. Urea transporters (efflux transporter) have been detected in sweat glands of skin [63], and its presence as a sweat metabolite possibly indicates the role of sweat glands in urea excretion. A detailed list of transporters is listed in Table 1.

## 4. The Implication of SLC and ABC Transporters in the Skin and Related Diseases

SLC and ABC transporters in the skin are understudied and relatively few reports are available on their relation to skin diseases. SLC36A1/PAT1 is responsible for the champagne dilution phenotype in horses characterized by dilution of hair color intensity in both red and black pigments. The color of the horse changes from chestnut to gold champagne, bay to amber champagne, and black to classic champagne [65]. Lactate is a metabolite found in sweat, which is a breakdown product of glucose. The transport of lactate is mediated by SLC16 (monocarboxylate transporters, MCT) expressed in the skin [66]. When studied with chicken choroid explants for wound healing, MCT3 has been reported to be lost, while increased expression of MCT4 was observed following a scratch [67]. Furthermore, a minor deposition of lactate in the cells and tissue under oxygenated condition is itself sufficient for wound healing with the full sequence of vasculogenesis and collagen deposition phase. Lactate enhances the set of activities for wound healing as endothelial cell mobility, increased collagen synthesis and its post-transitional modification and deposition and cell proliferation [68]. Furthermore, GLUT1 has been shown to be a promising drug target to treat psoriasis. Deletion of GLUT1 decreased hyperplasia in mice and topical application of GLUT1 inhibitor reduced inflammation in psoriatic organoids models. GLUT1 was reported to be required by keratinocytes for injury/inflammation associated proliferation [69]. Identification of urea transporters in the skin opens many possibilities to treat uremic patients by creating a “sweating kidney” as they have increased expression levels of SLC14A1–2 transporters in sweat glands. Contrary to these findings, urea transporters are involved in the uremic frost as well. Therefore, there is an increasing need to study these specific transporters in detail using suitable ex-vivo, in-vivo, and in-vitro models [70].

Similarly, *ABCC6* is implicated with a rare genetic recessive disorder pseudoxanthoma elasticum resulting in mineralization of elastic tissues, including skin [71]. The real substrate for ABCC6 is still unknown and although localized in mitochondria-associated membrane [51,72]. Wounds are again another area related to the skin where minimal information is available in connection to skin SLC and ABC transporters. Post-injury, the 4-stage healing process (hemostasis, inflammation, proliferation, and remodeling) begins naturally and many biochemicals, such as growth factors, chemokines, cytokines, extracellular matrix and regulatory molecules, participate in the process [73,74]. Arginine plays an important role in wound healing, being a precursor to proline and ornithine, which are responsible for the synthesis of nitric oxide and collagen. Nitric oxide under oxygenated conditions creates the reactive oxygen species required for gene expression and cellular differentiation. Inhibition of nitric oxide resulted in decreased collagen synthesis and impaired wound healing [75]. *ABCG2* expression is required for proper expansion and differentiation of epidermal stem cells (reside in a bulge of a hair follicle). *ABCG’s* knockouts resulted in elevated levels of reactive oxygen species and damage DNA leading to proliferation arrest [76].

## 5. Conclusions

The area of transporters and solute carriers, in general, is under-investigated as compared to other classes of membrane proteins and even less studied in the skin. Currently, the approaches utilized are either based on mRNA measurements (>80% of times) and direct proteins measurements (<20%) using mass spectrometry. There is an utmost need to utilize both methods together and correlate them to total skin and sweat metabolites to gain a better understanding of physiological processes occurring in the skin. This will not only enable the scientists to understand the pathophysiology of skin diseases better but also aid in developing suitable formulations for topical delivery of pharmaceuticals.

## Figures and Tables

**Figure 5:**
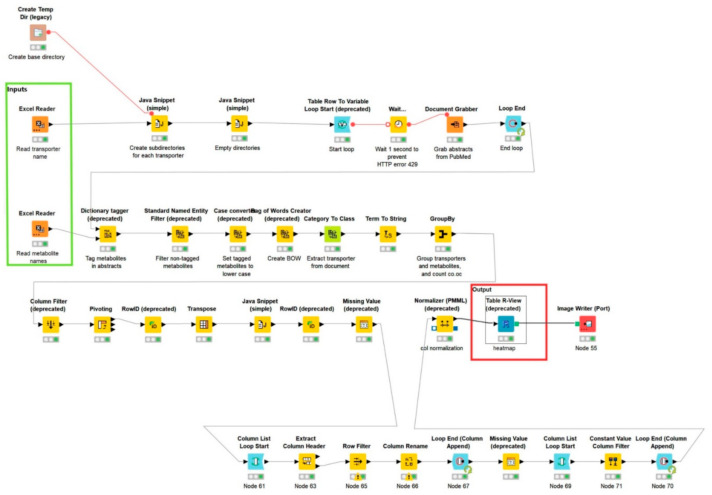
KNIME workflow used to map the co-occurrences of sweat metabolites vs. skin SLC and ABC transporters. Customized from original. https://www.knime.com/term-coocurrence-heatmap-example (accessed on 15 March 2021).

**Table 1:** List of transporters present in skin, including whether they were identified by mRNA-based methods or MS-based proteomics (marked with *).

S.No	Transporters (In Skin)	Detection		Refs
		mRNA	Proteomics	
1	SLC28A3	*		[37]
	SLCO2B1,SLCO1B1,SLCO1B2,SLCO1C1,SLCO3A1,SLCO4A1,SLCO4C1	*		[37]
	SLC16A1,SLC16A4	*		[37]
	SLC19A1	*		[37]
2	ABCA5,ABCA2,ABCA6,ABCA9,ABCA10,ABCA12	*		[36]
	ABCB3,ABCB2,ABCB4,ABCB6,ABCB7,ABCB8,ABCB10,ABCB11	*		[36]
	ABCC1,ABCC2,ABCC3,ABCC4,ABCC5,ABCC7,ABCC9,ABCC10,ABCC11	*		[36]
	ABCD1,ABCD2,ABCD3,ABCD4	*		[36]
	ABCE1	*		[36]
	ABCF1,ABCF2,ABCF3	*		[36]
	ABCG1,ABCG4	*		[36]
3	ABCC1(DOMINANT)	*		[49]
	ABCG2,ABCB1,ABCC2 (relatively less)	*		[49]
4	ABCC3 (most abundant)	*		[35]
	ABCB1,ABCA7,ABCG2 (less abundant)	*		[35]
	ABCA2,ABCC1 (moderately abundant)	*		[35]
	SLC22A3(most abundant)	*		[35]
	SLCO3A1,SLC16A7,SLCO2B1(moderately abundant)	*		[35]
5	ABCA6,ABCA8		*	[38]
	ABCB2,ABCB3,ABCB6		*	[38]
	ABCC12		*	[38]
	ABCD3		*	[38]
	ABCE1		*	[38]
6	SLC14 (1 and 2)	*		[63]
7	SLC47A1 and SLC47A2	*		[64]

## Data Availability

The KNIME workflow is available from the corresponding author upon request.

## References

[1] Tsuruta D., Green K.J., Getsios S., Jones J.C.R. (2002). The barrier function of skin: How to keep a tight lid on water loss. Trends Cell Biol..

[2] Wilke K., Martin A., Terstegen L., Biel S.S. (2007). A short history of sweat gland biology. Int. J. Cosmet. Sci..

[3] Groscurth P. (2002). Anatomy of sweat glands. Curr. Probl. Dermatol..

[4] Hodge B.D., Sanvictores T., Brodell R.T. (2021). Anatomy, Skin Sweat Glands.

[5] Sato K., Leidal R., Sato F. (1987). Morphology and development of an apoeccrine sweat gland in human axillae. Am. J. Physiol..

[6] Sato K., Sato F. (1987). Sweat secretion by human axillary apoeccrine sweat gland in vitro. Am. J. Physiol..

[7] Baker L.B. (2019). Physiology of sweat gland function: The roles of sweating and sweat composition in human health. Temperature.

[8] Sato K., Kang W.H., Saga K., Sato K.T. (1989). Biology of sweat glands and their disorders. I. Normal sweat gland function. J. Am. Acad. Dermatol..

[9] Jadoon S., Karim S., Akram M.R., Kalsoom Khan A., Zia M.A., Siddiqi A.R., Murtaza G. (2015). Recent developments in sweat analysis and its applications. Int. J. Anal. Chem..

[10] Saint-Criq V., Gray M.A. (2017). Role of CFTR in epithelial physiology. Cell. Mol. Life Sci..

[11] Kell D.B. (2015). What would be the observable consequences if phospholipid bilayer diffusion of drugs into cells is negligible?. Trends Pharm. Sci..

[12] Kell D.B. (2015). The transporter-mediated cellular uptake of pharmaceutical drugs is based on their metabolite-likeness and not on their bulk biophysical properties: Towards a systems pharmacology. Perspect. Sci..

[13] Kell D.B. (1992). Transport—Linked Phosphorylation: Problems and Prospects. Curr. Top. Cell. Regul..

[14] Kell D.B., Dobson P.D., Bilsland E., Oliver S.G. (2013). The promiscuous binding of pharmaceutical drugs and their transporter-mediated uptake into cells: What we (need to) know and how we can do so. Drug Discov. Today.

[15] Kell D.B., Dobson P.D., Oliver S.G. (2011). Pharmaceutical drug transport: The issues and the implications that it is essentially carrier-mediated only. Drug Discov. Today.

[16] Kell D.B., Oliver S.G. (2014). How drugs get into cells: Tested and testable predictions to help discriminate between transporter-mediated uptake and lipoidal bilayer diffusion. Front. Pharm..

[17] Alexander S.P.H., Kelly E., Mathie A., Peters J.A., Veale E.L., Armstrong J.F., Faccenda E., Harding S.D., Pawson A.J., Sharman J.L. (2019). THE CONCISE GUIDE TO PHARMACOLOGY 2019/20: Transporters. Br. J. Pharm..

[18] Babcock J.J., Li M. (2014). Deorphanizing the human transmembrane genome: A landscape of uncharacterized membrane proteins. Acta Pharm. Sin..

[19] Dean M., Hamon Y., Chimini G. (2001). The human ATP-binding cassette (ABC) transporter superfamily. Genome Res..

[20] Hediger M.A., Romero M.F., Peng J.-B., Rolfs A., Takanaga H., Bruford E.A. (2004). The ABCs of solute carriers: Physiological, pathological and therapeutic implications of human membrane transport proteinsIntroduction. Pflugers Arch..

[21] Borst P., Zelcer N., Van Helvoort A. (2000). ABC transporters in lipid transport. Biochim. Biophys Acta..

[22] Ruetz S., Gros P. (1994). Phosphatidylcholine translocase: A physiological role for the mdr2 gene. Cell.

[23] Schmitz G., Kaminski W.E., Orsó E. (2000). ABC transporters in cellular lipid trafficking. Curr. Opin. Lipidol..

[24] Schmitz G., Langmann T., Heimerl S. (2001). Role of ABCG1 and other ABCG family members in lipid metabolism. J. Lipid. Res..

[25] Lu K., Lee M.H., Patel S.B. (2001). Dietary cholesterol absorption; more than just bile. Trends Endocrinol. Metab..

[26] Seguin A., Ward D.M. (2018). Mitochondrial ABC Transporters and Iron Metabolism. Int. J. Clin. Exp. Pathol..

[27] Vasiliou V., Vasiliou K., Nebert D.W. (2009). Human ATP-binding cassette (ABC) transporter family. Hum. Genom..

[28] Glavinas H., Krajcsi P., Cserepes J., Sarkadi B. (2005). The Role of ABC Transporters in Drug Resistance, Metabolism and Toxicity. Curr. Drug Deliv..

[29] Perland E., Fredriksson R. (2017). Classification Systems of Secondary Active Transporters. Trends Pharm. Sci..

[30] Lin L., Yee S.W., Kim R.B., Giacomini K.M. (2015). SLC transporters as therapeutic targets: Emerging opportunities. Nat. Rev. Drug Discov..

[31] Zhang Y., Zhang Y., Sun K., Meng Z., Chen L. (2018). The SLC transporter in nutrient and metabolic sensing, regulation, and drug development. J. Mol. Cell Biol..

[32] El-Awady R., Saleh E., Hashim A., Soliman N., Dallah A., Elrasheed A., Elakraa G. (2017). The Role of Eukaryotic and Prokaryotic ABC Transporter Family in Failure of Chemotherapy. Front. Pharm..

[33] Kottra G., Daniel H. (2001). Bidirectional electrogenic transport of peptides by the proton-coupled carrier PEPT1 in Xenopus laevis oocytes: Its asymmetry and symmetry. J. Physiol..

[34] Wishart D.S., Feunang Y.D., Marcu A., Guo A.C., Liang K., Vazquez-Fresno R., Sajed T., Johnson D., Li C., Karu N. (2018). HMDB 4.0: The human metabolome database for 2018. Nucleic Acids Res..

[35] Takechi T., Hirota T., Sakai T., Maeda N., Kobayashi D., Ieiri I. (2018). Interindividual differences in the expression of atp-binding cassette and solute carrier family transporters in human skin: Dna methylation regulates transcriptional activity of the human abcc3 gene. Drug Metab. Dispos..

[36] Takenaka S., Itoh T., Fujiwara R. (2013). Expression pattern of human ATP-binding cassette transporters in skin. Pharm. Res. Perspect..

[37] Fujiwara R., Takenaka S., Hashimoto M., Narawa T., Itoh T. (2014). Expression of human solute carrier family transporters in skin: Possible contributor to drug-induced skin disorders. Sci. Rep..

[38] Al-Majdoub Z.M., Achour B., Couto N., Howard M., Elmorsi Y., Scotcher D., Alrubia S., El-Khateeb E., Vasilogianni A.-M., Alohali N. (2020). Mass spectrometry-based abundance atlas of ABC transporters in human liver, gut, kidney, brain and skin. FEBS Lett..

[39] Aebersold R., Mann M. (2016). Mass-spectrometric exploration of proteome structure and function. Nature.

[40] Larance M., Lamond A.I. (2015). Multidimensional proteomics for cell biology. Nat. Rev. Mol. Cell Biol..

[41] Dyring-Andersen B., Løvendorf M.B., Coscia F., Santos A., Møller L.B.P., Colaço A.R., Niu L., Bzorek M., Doll S., Andersen J.L. (2020). Spatially and cell-type resolved quantitative proteomic atlas of healthy human skin. Nat. Commun..

[42] Wang D., Eraslan B., Wieland T., Hallström B., Hopf T., Zolg D.P., Zecha J., Asplund A., Li L.-h., Meng C. (2019). A deep proteome and transcriptome abundance atlas of 29 healthy human tissues. Mol. Syst. Biol..

[43] Couto N., Newton J.R.A., Russo C., Karunakaran E., Achour B., Al-Majdoub Z.M., Sidaway J., Rostami-Hodjegan A., Clench M.R., Barber J. (2020). Label-free Quantitative Proteomics and Substrate Based Mass Spectrometry Imaging of Xenobiotic Metabolizing Enzymes in ex Vivo Human Skin and a Human Living Skin Equivalent Model. Drug Metab. Dispos..

[44] Bliss E., Heywood W.E., Benatti M., Sebire N.J., Mills K. (2016). An optimised method for the proteomic profiling of full thickness human skin. Biol. Proced. Online.

[45] Hughes C.S., Foehr S., Garfield D.A., Furlong E.E., Steinmetz L.M., Krijgsveld J. (2014). Ultrasensitive proteome analysis using paramagnetic bead technology. Mol. Syst. Biol..

[46] Kaleja P., Emmert H., Gerstel U., Weidinger S., Tholey A. (2020). Evaluation and improvement of protein extraction methods for analysis of skin proteome by noninvasive tape stripping. J. Proteom..

[47] O’Hagan S., Kell D.B. (2015). Software review: The KNIME workflow environment and its applications in genetic programming and machine learning. Genet. Program. Evolvable. Mach..

[48] Fillbrunn A., Dietz C., Pfeuffer J., Rahn R., Landrum G.A., Berthold M.R. (2017). KNIME for reproducible cross-domain analysis of life science data. J. Biotechnol..

[49] Osman-Ponchet H., Boulai A., Kouidhi M., Sevin K., Alriquet M., Gaborit A., Bertino B., Comby P., Ruty B. (2014). Characterization of ABC transporters in human skin. Drug Metabol. Drug Interact..

[50] Okamura N., Sakaeda T., Okumura K. (2004). Pharmacogenomics of MDR and MRP subfamilies. Pers. Med..

[51] Hendig D., Langmann T., Fau-Kocken S., Kocken S., Fau-Zarbock R., Zarbock R., Fau-Szliska C., Szliska C., Fau-Schmitz G., Schmitz G. (2008). Gene expression profiling of ABC transporters in dermal fibroblasts of pseudoxanthoma elasticum patients identifies new candidates involved in PXE pathogenesis. Lab. Investig..

[52] Boll M., Daniel H., Gasnier B. (2004). The SLC36 family: Proton-coupled transporters for the absorption of selected amino acids from extracellular and intracellular proteolysis. Pflug. Arch..

[53] Shen L., Qian C., Cao H., Wang Z., Luo T., Liang C. (2018). Upregulation of the solute carrier family 7 genes is indicative of poor prognosis in papillary thyroid carcinoma. World J. Surg. Oncol..

[54] Wright E.M. (2013). Glucose transport families SLC5 and SLC50. Mol. Asp. Med..

[55] Wright E.M., Turk E. (2004). The sodium/glucose cotransport family SLC5. Pflugers Arch..

[56] Romero M.F., Fulton C.M., Boron W.F. (2004). The SLC4 family of HCO_3_^-^ transporters. Mol. Asp. Med..

[57] Oh C.S., Hong J.H., Jin S.N., Lee W.J., Lee Y.S., Lee E. (2017). Expression of glucose transporters in the developing rat skin. Anat. Cell Biol..

[58] Gherzi R., Melioli G., de Luca M., D’Agostino A., Distefano G., Guastella M., D’Anna F., Franzi A.T., Cancedda R. (1992). “HepG2/erythroid/brain” type glucose transporter (GLUT1) is highly expressed in human epidermis: Keratinocyte differentiation affects glut1 levels in reconstituted epidermis. J. Cell. Physiol..

[59] Bonen A., Heynen M., Hatta H. (2006). Distribution of monocarboxylate transporters MCT1-MCT8 in rat tissues and human skeletal muscle. Appl. Physiol. Nutr. Metab..

[60] Cibrian D., Castillo-González R., Fernández-Gallego N., de la Fuente H., Jorge I., Saiz M.L., Punzón C., Ramírez-Huesca M., Vicente-Manzanares M., Fresno M. (2020). Targeting L-type amino acid transporter 1 in innate and adaptive T cells efficiently controls skin inflammation. J. Allergy Clin. Immunol..

[61] Schnorr O., Suschek C.V., Kolb-Bachofen V. (2003). The importance of cationic amino acid transporter expression in human skin. J. Investig. Derm..

[62] Jensen A., Figueiredo-Larsen M., Holm R., Broberg M.L., Brodin B., Nielsen C.U. (2014). PAT1 (SLC36A1) shows nuclear localization and affects growth of smooth muscle cells from rats. Am. J. Physiol. Endocrinol. Metab..

[63] Xie L., Jin L., Feng J., Lv J. (2017). The expression of AQP5 and UTs in the sweat glands of uremic patients. BioMed Res. Int..

[64] Alriquet M., Sevin K., Gaborit A., Comby P., Ruty B., Osman-Ponchet H. (2015). Characterization of SLC transporters in human skin. Admet Dmpk.

[65] Cook D., Brooks S Fau-Bellone R., Bellone R Fau-Bailey E., Bailey E. (2008). Missense mutation in exon 2 of SLC36A1 responsible for champagne dilution in horses. PLoS Genet..

[66] Gallagher S.M., Castorino J.J., Philp N.J. (2009). Interaction of monocarboxylate transporter 4 with beta1-integrin and its role in cell migration. Am. J. Physiol. Cell Physiol..

[67] Gallagher-Colombo S., Maminishkis A., Fau-Tate S., Tate S., Fau-Grunwald G.B., Grunwald G.B., Fau-Philp N.J., Philp N.J. (2010). Modulation of MCT3 expression during wound healing of the retinal pigment epithelium. Investig. Ophthalmol. Vis. Sci..

[68] Porporato P.E., Payen V.L., De Saedeleer C.J., Préat V., Thissen J.-P., Feron O., Sonveaux P. (2012). Lactate stimulates angiogenesis and accelerates the healing of superficial and ischemic wounds in mice. Angiogenesis.

[69] Zhang Z., Zi Z., Lee E.E., Zhao J., Contreras D.C., South A.P., Abel E.D., Chong B.F., Vandergriff T., Hosler G.A. (2018). Differential glucose requirement in skin homeostasis and injury identifies a therapeutic target for psoriasis article. Nat. Med..

[70] Klein J.D., Sands J.M. (2016). Urea transport and clinical potential of urearetics. Curr. Opin. Nephrol. Hypertens.

[71] Váradi A., Szabó Z., Pomozi V., de Boussac H., Fülöp K., Arányi T. (2011). ABCC6 as a target in pseudoxanthoma elasticum. Curr. Drug Targets.

[72] Martin L.J., Lau E., Singh H., Vergnes L., Tarling E.J., Mehrabian M., Mungrue I., Xiao S., Shih D., Castellani L. (2012). ABCC6 localizes to the mitochondria-associated membrane. Circ. Res..

[73] Cañedo-Dorantes L., Cañedo-Ayala M. (2019). Skin Acute Wound Healing: A Comprehensive Review. Int. J. Inflam..

[74] Gomes A., Teixeira C., Ferraz R., Prudêncio C., Gomes P. (2017). Wound-Healing Peptides for Treatment of Chronic Diabetic Foot Ulcers and Other Infected Skin Injuries. Molecules.

[75] Witte M.B., Barbul A. (2003). Arginine physiology and its implication for wound healing. Wound Repair Regen..

[76] Chang H.-M., Huang W.-Y., Lin S.-J., Huang W.-C., Shen C.-R., Mao W.-Y., Shen C.-N. (2016). ABCG2 deficiency in skin impairs re-epithelialization in cutaneous wound healing. Exp. Derm..

